# Sequencing of *E. coli* strain UTI89 on multiple sequencing platforms

**DOI:** 10.1186/s13104-020-05335-4

**Published:** 2020-10-20

**Authors:** Shannon N. Fenlon, Yuemin Celina Chee, Jacqueline Lai Yuen Chee, Yeen Hui Choy, Alexis Jiaying Khng, Lu Ting Liow, Kurosh S. Mehershahi, Xiaoan Ruan, Stephen W. Turner, Fei Yao, Swaine L. Chen

**Affiliations:** 1 Genome Institute of Singapore, 60 Biopolis Street, Genome, #02-01, Singapore, 138672 Singapore; 2 Department of Medicine, Yong Loo Lin School of Medicine, National University of Singapore, 1E Kent Ridge Road, NUHS Tower Block, Level 10, Singapore, 119228 Singapore; 3 Pacific Biosciences, 1305 O’Brien Dr, Menlo Park, CA 94025 USA

**Keywords:** *Escherichia coli*, UPEC, Urinary Tract Infection (UTI), Ion Torrent, SOLiD, Illumina, Oxford Nanopore, MinION, PacBio, Roche454

## Abstract

**Objectives:**

The availability of matched sequencing data for the same sample across different sequencing platforms is a necessity for validation and effective comparison of sequencing platforms. A commonly sequenced sample is the lab-adapted MG1655 strain of *Escherichia coli*; however, this strain is not fully representative of more complex and dynamic genomes of pathogenic *E. coli* strains.

**Data description:**

We present six new sequencing data sets for another *E. coli* strain, UTI89, which is an extraintestinal pathogenic strain isolated from a patient suffering from a urinary tract infection. We now provide matched whole genome sequencing data generated using the PacBio RSII, Oxford Nanopore MinION R9.4, Ion Torrent, ABI SOLiD, and Illumina NextSeq sequencers. Together with other publically available datasets, UTI89 has a nearly complete suite of data generated on most second- and third-generation sequencers. These data can be used as an additional validation set for new sequencing technologies and analytical methods. More than being another *E. coli* strain, however, UTI89 is pathogenic, with a 10% larger genome, additional pathogenicity islands, and a large plasmid, features that are common among other naturally occurring and disease-causing *E. coli* isolates. These data therefore provide a more medically relevant test set for development of algorithms.

## Objective

Control sequencing data across different sequencing platforms is extremely important for validation and effective comparison of sequencing platforms. A commonly sequenced sample that has been extensively used for these purposes is the MG1655 strain of *E. coli* [1]. However, the MG1655 genome is smaller and less complex than those of some pathogenic *E. coli* strains [2, 3]. As part of control experiments, we have sequenced UTI89, a uropathogenic *E. coli* (UPEC) strain originally isolated from a patient suffering from an acute bladder infection [4], using several different sequencing technologies, including ABI SOLiD, Ion Torrent, PacBio, Oxford Nanopore, and Illumina. Our new data supplements previously published sequencing data generated using the Roche 454 [4], Illumina HiSeq [5], and the original Oxford Nanopore Technologies MinION [6]. With the inclusion of these new data sets, *E. coli* strain UTI89 now has a nearly complete set of raw sequence data generated using most second- and third-generation sequencers. For some of the technologies we have multiple data sets, such as for PacBio, which spans the first iteration of the RSII sequencing chemistry (XL/C2) in 2012 up to the P6-C4 chemistry (which was current in 2018), which led to a more than fivefold increase in mean read length.

## Data description

The new data sets are summarized in Table 1. Details of library preparation and sequencing methods for the new datasets are presented below.

**Table 1:** Overview of data sets

Label	Name of data file/data set	File types (file extension)	Data repository and identifier (DOI or accession number)
Data set 1	New UTI89 genomic data	XLSX (containing data on FASTQ files)	Figshare (10.6084/m9.figshare.12195663)
Data set 2	Previously published UTI89 genomic data	XLSX (containing data on FASTQ files)	Figshare (10.6084/m9.figshare.12195675)
Applied Biosystems SOliD 3 (new)	UTI89 – SoliD 3 LMP	FASTQ	NCBI Sequence Read Archive (https://identifiers.org/ncbi/insdc.sra:SRX4387579 [7]; https://identifiers.org/ncbi/insdc.sra:SRR7517573 [8]; https://identifiers.org/ncbi/insdc.sra:SRR8247388 [9])
Ion Torrent PGM (new)	UTI89/pBAD33 – IonTorrent	FASTQ	NCBI Sequence Read Archive (https://identifiers.org/ncbi/insdc.sra:SRX4225380 [11]; https://identifiers.org/ncbi/insdc.sra:SRR7352157 [12])
Pacific Biosciences RSII (XL/C2) (new)	SLC-66 – PacBio XL/C2	FASTQ	NCBI Sequence Read Archive (https://identifiers.org/ncbi/insdc.sra:SRX4387449 [13]; https://identifiers.org/ncbi/insdc.sra:SRR7517443 [14]; https://identifiers.org/ncbi/insdc.sra:SRR7525090 [15])
Illumina NextSeq 500 (new)	UTI89 – NextSeq 500	FASTQ	NCBI Sequence Read Archive (https://identifiers.org/ncbi/insdc.sra:SRX4223297 [16]; https://identifiers.org/ncbi/insdc.sra:SRR7349974 [17])
Oxford Nanopore MinION Mk 1b FLO-MIN106 (R9.4) (new)	UTI89 – MinION R9.4	FASTQ	NCBI Sequence Read Archive (https://identifiers.org/ncbi/insdc.sra:SRX4387499 [18]; https://identifiers.org/ncbi/insdc.sra:SRR7517493 [19])
Pacific Biosciences RSII (P6-C4) (new)	UTI89 – PacBio P6-C4	FASTQ	NCBI Sequence Read Archive (https://identifiers.org/ncbi/insdc.sra:SRX5058882 [20]; https://identifiers.org/ncbi/insdc.sra:SRX5058883 [21]; https://identifiers.org/ncbi/insdc.sra:SRR8240630 [22]; https://identifiers.org/ncbi/insdc.sra:SRR8240631 [23])
Roche 454 (previous) [4]	UTI89 - 454	FASTQ	NCBI Sequence Read Archive (https://identifiers.org/ncbi/insdc.sra:SRX000179 [24]; https://identifiers.org/ncbi/insdc.sra:SRR000868 [25]; https://identifiers.org/ncbi/insdc.sra:SRR000869 [26]; https://identifiers.org/ncbi/insdc.sra:SRR000870 [27]; https://identifiers.org/ncbi/insdc.sra:SRR000871 [28]; https://identifiers.org/ncbi/insdc.sra:SRR000872 [29]; https://identifiers.org/ncbi/insdc.sra:SRR000873 [30])
Illumina Hiseq 2000 (previous) [5]	UTI89 – HiSeq 2000	FASTQ	NCBI Sequence Read Archive (https://identifiers.org/ncbi/insdc.sra:ERX632843 [31]; https://identifiers.org/ncbi/insdc.sra:ERX632844 [32]; https://identifiers.org/ncbi/insdc.sra:ERR687900 [33]; https://identifiers.org/ncbi/insdc.sra:ERR687901 [34])
Oxford Nanopore MinION R7 (previous) [6]	UTI89 – MinION R7	FASTQ	NCBI Sequence Read Archive (https://identifiers.org/ncbi/insdc.sra:ERX987748 [35]; https://identifiers.org/ncbi/insdc.sra:ERR908493 [36])

### SOLiD

#### Library preparation

Genomic DNA was extracted from UTI89 grown overnight in Lysogeny Broth (LB) and used to generate Long Mate Pair (LMP) libraries. LMP libraries were generated using an insert size of 3–4 kb according to the manufacturer’s instructions to produce a 375 bp library.

#### Sequencing

A 2x35bp LMP sequencing run was performed on two spots of an 8 spot slide using the Applied Biosystems SOLiD3 platform [7–9].

### Ion Torrent

#### Library preparation

Genomic DNA was extracted from UTI89 harbouring the pBAD33 plasmid [10] grown overnight in LB. Sequencing libraries were then generated using the Ion Xpress™ Plus gDNA library preparation protocol according to the manufacturer’s instructions.

#### Sequencing

A 200 bp sequencing run was performed on the personal genome machine (PGM) system using the Ion PGM™ 200 Sequencing Kit with a 316 chip [11, 12].

### PacBio, RSII, XL/C2 Chemistry

#### Library preparation

Genomic DNA was extracted from SLC-66 (UTI89 with a kanamycin cassette integrated into the phage HK022 integration site) grown overnight in LB. Large insert (15 Kb) native SMRTbell sequencing libraries were generated according to the manufacturer’s protocols.

#### Sequencing

Sequencing was performed on 6 SMRT Cells using XL/C2 Sequencing chemistry [13–15].

### Illumina

#### Library preparation

Genomic DNA was extracted from UTI89 grown overnight in LB. Sequencing libraries were built using the Illumina TruSeq Nano DNA LT kit according to the manufacturer’s instructions, with shearing to 350 bp.

#### Sequencing

A 2x150bp sequencing run was performed using the Illumina NextSeq 500 and a NextSeq Mid Output flow cell and reagents [16, 17].

### Oxford Nanopore, MinION Mk1B Device, R9.4, 1D Ligation sequencing

#### Library preparation

Genomic DNA was extracted from UTI89 grown overnight in LB. 1 μg of unsheared DNA was used to prepare sequencing libraries using the Ligation sequencing kit 1D R9 version (SQK-LSK108) according to the manufacturer’s instructions.

#### Sequencing

The prepared sequencing library was loaded onto a FLO-MIN106 R9.4 with Spot-ON and a 24 h sequencing run was performed. Base calling was subsequently performed using Oxford Nanopore’s Albacore Sequencing Pipeline Software (version 1.2.1) [18, 19].

### PacBio, RSII, P6-C4 Chemistry

#### Library preparation

Genomic DNA was extracted from UTI89 grown overnight in LB. Large insert (20 Kb) native SMRTbell sequencing libraries were generated according to the manufacturer’s instructions.

#### Sequencing

Sequencing was performed on 2 SMRT Cells using P6-C4 Sequencing chemistry [20–23].

### Previously published data sets

There are three previously published data sets generated using other sequencing platforms or sequencer versions: Roche 454 [4, 24–30], Illumina HiSeq 2000 [5, 31–34], and the original Oxford Nanopore MinION with an R7 flow cell [6, 35, 36]. The data presented in this manuscript complements these published datasets (also included in Table 1).

## Limitations

The following are limitations of these data:

1. The data was collected over a period of several years, and thus all experimental steps were performed by different persons.
2. Some strains contain plasmids or other markers (see details above).
3. Not every generation of sequencing machine or library preparation method was used.

## Data Availability

The data described in this Data note can be freely and openly accessed on Genbank. Please see Table 1 for accession numbers. Specifically, the experiment accessions for the newly presented data are: SRX4387579 [7], SRX4225380 [11], SRX4387449 [13], SRX4223297 [16], SRX4387499 [18], SRX5058882 [20], and SRX5058883 [21]. The experiment accession for the previously published data are: SRX000179 [24], ERX632843 [31], ERX632844 [32], and ERX987748 [35].

## Abbreviations

UPEC: Uropathogenic *Escherichia coli*
UTI: Urinary tract infection
LB: Lysogeny broth
LMP: Long mate pair
PGM: Personal genome machine
